# Supplementary material for: Neurophysiological signatures of cortical micro-architecture
Source: bioRxiv. 2023 Jan 23:2023.01.23.525101. Preprint. [Version 1] doi: 10.1101/2023.01.23.525101 (PMC9900796; doi:10.1101/2023.01.23.525101)
Supplement: 1 [file NIHPP2023.01.23.525101V1-supplement-1.pdf]

bioRxiv preprint doi: https://doi.org/10.1101/2023.01.23.525101; this version posted January 23, 2023. The copyright holder for this preprint (which was not certified by peer review) is the author/funder, who has granted bioRxiv a license to display the preprint in perpetuity. It is made available under aCC-BY 4.0 International license.

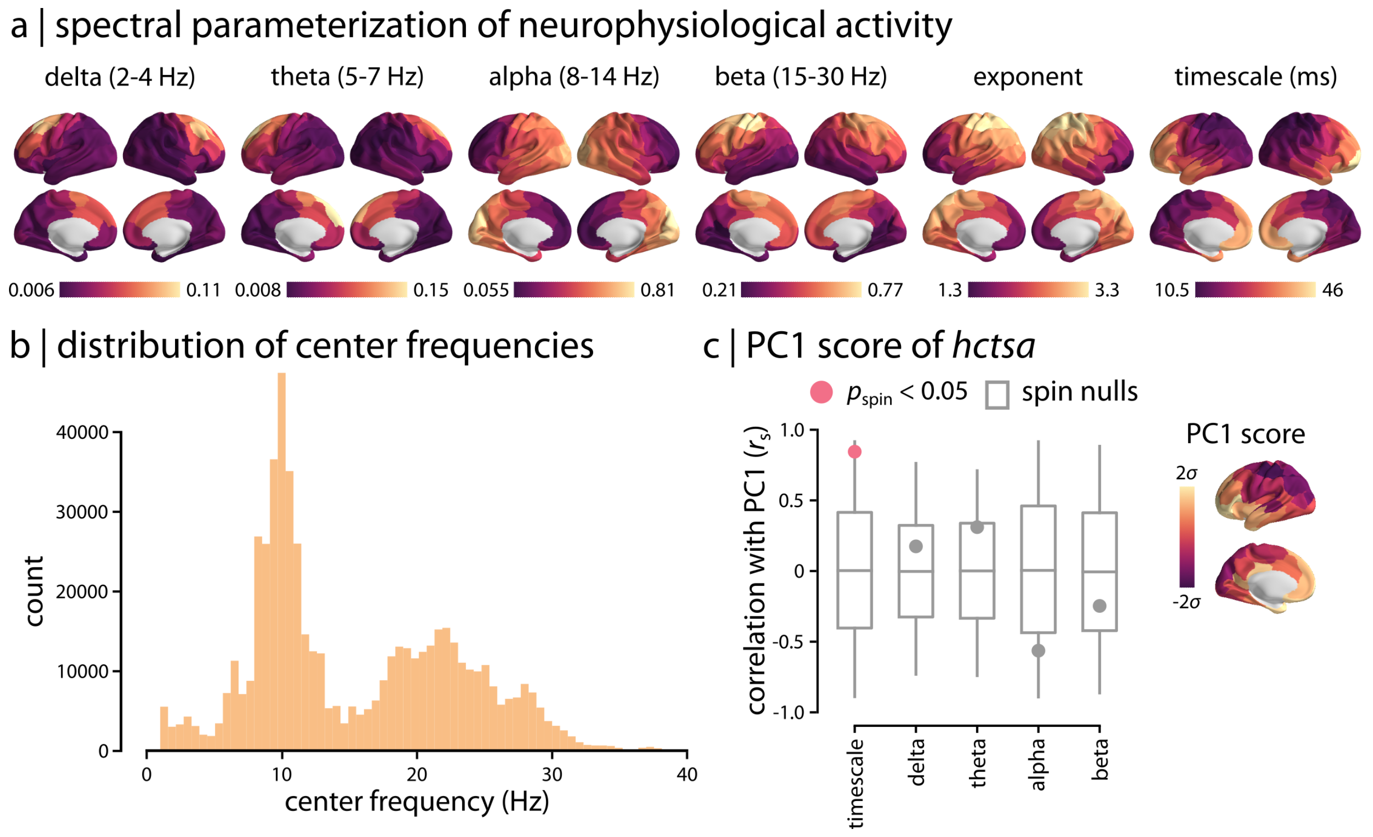

Figure S1. **Spectral parameterization of neurophysiological activity** | Spectral parameterization `FOOOF` toolbox was used to extract periodic and aperiodic components of the MEG power spectrum [21]. (a) The identified oscillatory peaks of the periodic component were used to estimate band-limited "oscillation score" maps at delta (2-4 Hz), theta (5-7 Hz), alpha (8-14 Hz), and beta (15-30 Hz) frequency bands. The oscillation scores reflect the average power at each region and frequency band, weighted by the probability of observing an oscillation peak at that region and band [21]. The aperiodic exponent and "knee" parameter (controls for the bend in the aperiodic component) were used to estimate intrinsic timescale (see *Methods* for details). Note that log-10 transformed intrinsic timescale map is depicted here for a more clear visualization. (b) Distribution of center frequencies of the identified periodic peaks are depicted across all vertices and participants. Visual inspection of the distribution shows clusters of peaks around the frequency bands shown in panel (a). (c) PC1 score map of `hctsa` time-series features was compared with the aperiodic-adjusted power maps and intrinsic timescale. Consistent with the results obtained with the total power maps at the canonical frequencies (Fig. 3), PC1 is significantly associated with the intrinsic timescale (FDR-corrected; 10 000 autocorrelation-preserving spin nulls). r_s denotes the Spearman's rank correlation coefficient.

bioRxiv preprint doi: https://doi.org/10.1101/2023.01.23.525101; this version posted January 23, 2023. The copyright holder for this preprint (which was not certified by peer review) is the author/funder, who has granted bioRxiv a license to display the preprint in perpetuity. It is made available under aCC-BY 4.0 International license.

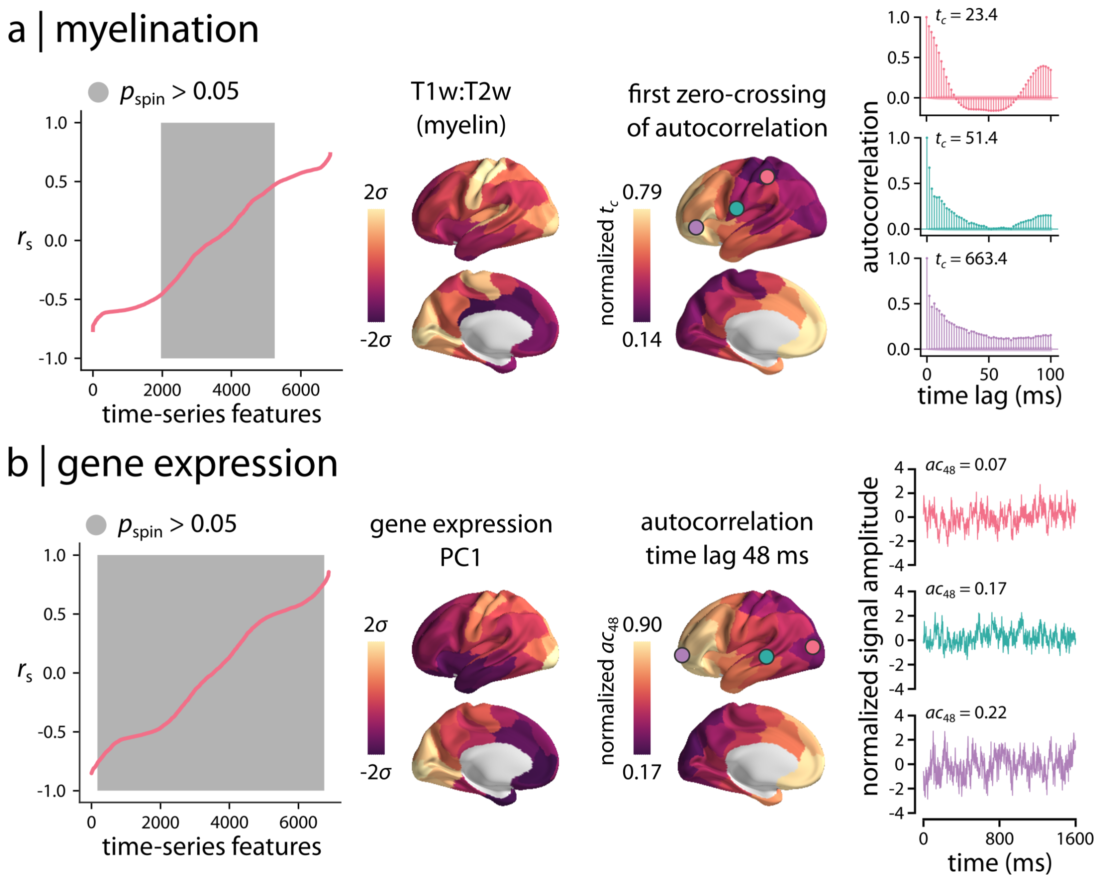

Figure S2. **Univariate analysis of neurophysiological time-series features** | Spearman's rank correlation coefficients (r_s) were used to investigate the univariate associations between `hctsa` time-series features of neurophysiological signal and two commonly-used micro-architectural maps: (a) T1w/Tw2 ratio as a proxy measure of myelination, and (b) principal component of gene expression. The resulting correlations were compared with null distributions of correlations obtained from 10 000 spatial autocorrelation-preserving nulls. Grey background indicates non-significant time-series features (FDR corrected). Examples of high loading time-series features are shown for each micro-architectural map. The group-average first zero-crossing time point of the autocorrelation function, t_c, is shown for T1w/T2w ratio. The group-average linear autocorrelation at a time lag of 48 ms, ac_{48}, is shown for principal component of gene expression. The autocorrelation function and short segments of raw time-series are also shown for a randomly selected participant at three different regions (circles on the brain surface: pink $\approx 5^{th}$ percentile, green $\approx 50^{th}$ percentile, purple $\approx 95^{th}$ percentile). Full lists of features, their correlation coefficients and p-values are available for T1w/T2w ratio and gene expression in the online Supplementary Files S5,6.

bioRxiv preprint doi: https://doi.org/10.1101/2023.01.23.525101; this version posted January 23, 2023. The copyright holder for this preprint (which was not certified by peer review) is the author/funder, who has granted bioRxiv a license to display the preprint in perpetuity. It is made available under aCC-BY 4.0 International license.

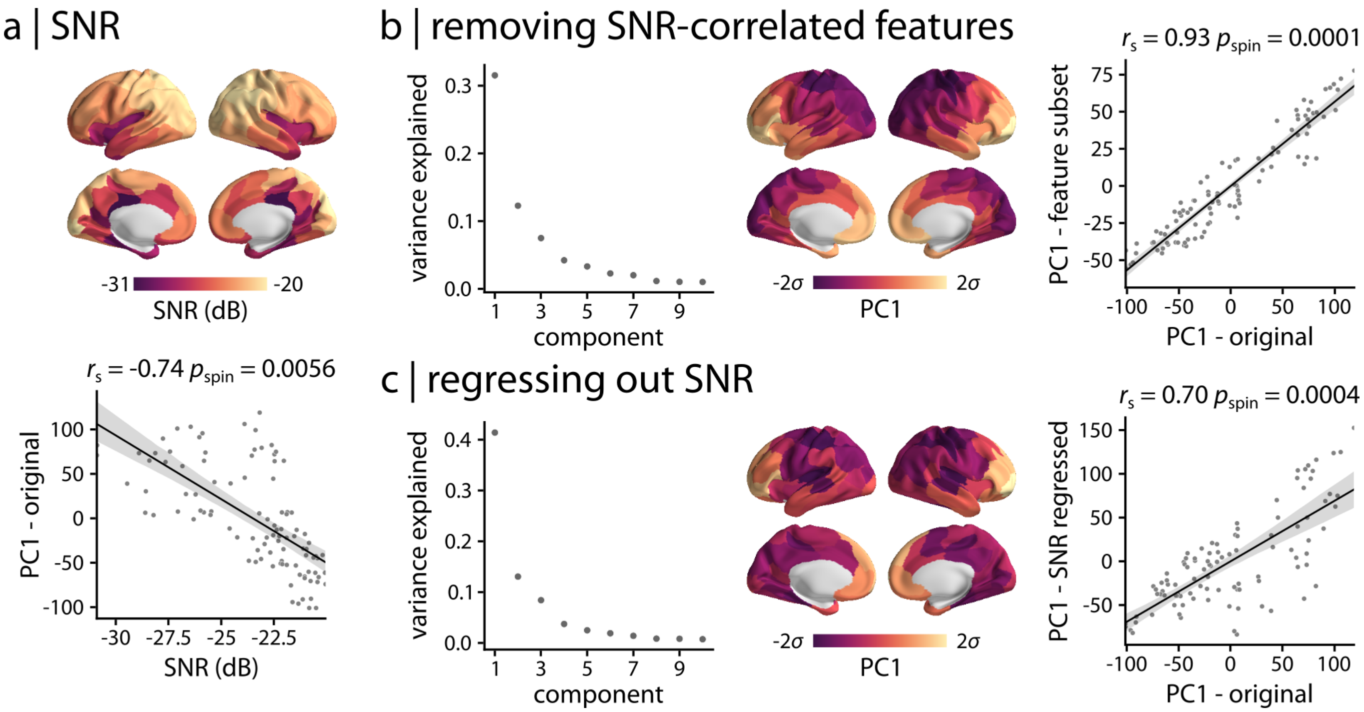

Figure S3. **Signal-to-noise ratio (SNR)** | (a) Source-level MEG signal-to-noise ratio (SNR) was estimated. Parcellated, group-average SNR map is depicted across the cortex. MEG SNR was compared with the principal component of neurophysiological dynamics (PC1 - original). (b) SNR was compared with full set of neurophysiological time-series features (i.e., 6 880 features) using univariate correlations. Features that were significantly correlated with SNR were removed without correcting for multiple comparisons ($p_{spin} < 0.05$, 10 000 spatial autocorrelation-preserving permutation tests) and PCA was repeated using the remaining 3 819 features. The principal component of the retained feature subset (PC1 - feature subset) explained 31.6% of the variance and was significantly correlated with the original PC1 from the full set of features. (c) SNR was linearly regressed out from the full set of time-series features. PCA was applied to the feature residuals. The principal component of SNR-regressed time-series features (PC1 - SNR regressed) explained 41.4% of the variance and was significantly correlated with the original PC1. r_s denotes the Spearman's rank correlation coefficient; linear regression lines are added to the scatter plots for visualization purposes only.

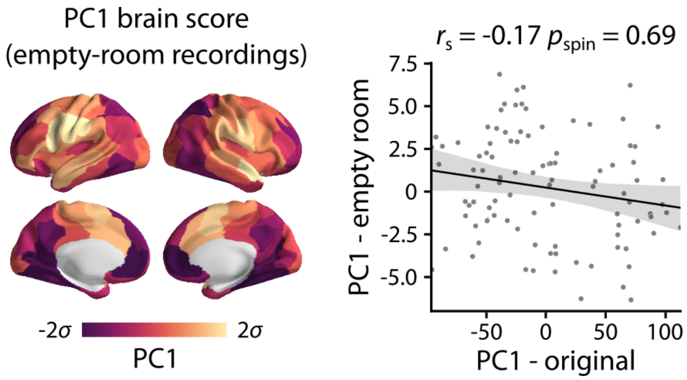

Figure S4. **Empty-room recordings** | PCA was applied to time-series features obtained from pre-processed empty-room MEG recordings. Following the Procrustes alignment of the resulting PCA weights with the PCA weights of the resting-state MEG recordings, the first principal components were compared between the two. The principal component of the empty-room time-series features (PC1 - empty room; variance explained = 41%) was not significantly correlated with the original PC1. r_s denotes the Spearman's rank correlation coefficient; linear regression line is added to the scatter plot for visualization purposes only.

bioRxiv preprint doi: https://doi.org/10.1101/2023.01.23.525101; this version posted January 23, 2023. The copyright holder for this preprint (which was not certified by peer review) is the author/funder, who has granted bioRxiv a license to display the preprint in perpetuity. It is made available under aCC-BY 4.0 International license.

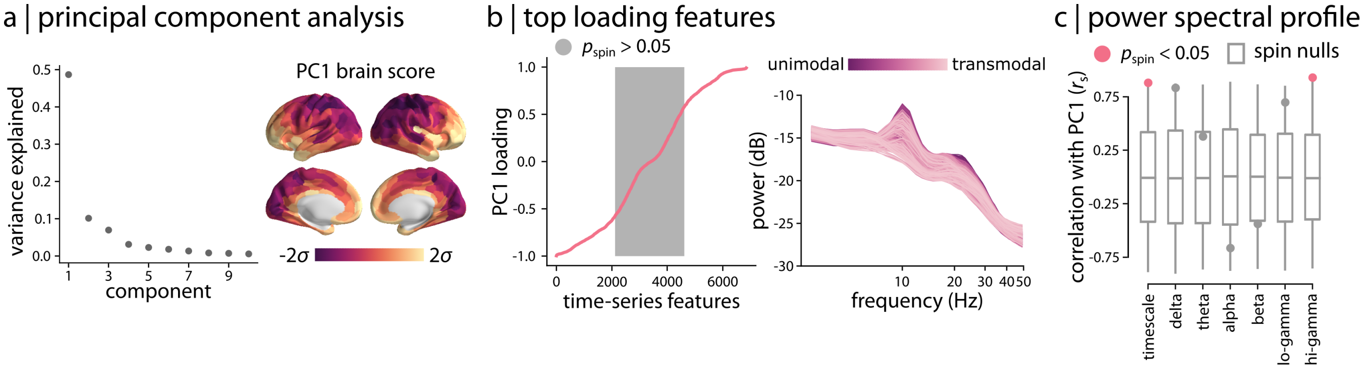

Figure S5. **Topographic distribution of neurophysiological dynamics for Schaefer-400 |** (a) Principal component analysis (PCA) was applied to MEG time-series features at a higher resolution parcellation (i.e., Schaefer-400 atlas). The first principal component accounted for 48.6% of the variance. The spatial organization of features captured by PC1 is depicted across the cortex, displaying a consistent pattern with the original PCA results obtained for the Schaefer-100 atlas (Fig. 3). (b) Top loading features contributing to PC1 were identified using Pearson correlation coefficients between PC1 pattern and all time-series features. Grey background indicates non-significant features based on 10 000 spatial autocorrelation-preserving permutation tests (FDR corrected). Consistent with the original analysis, the top loading features were mainly related to power spectral density. Regional power spectral densities are depicted, where each line represents a brain region. Regions are coloured by their position in the putative unimodal–transmodal hierarchy [72]. The full list of features, their loadings and p-values are available in the online Supplementary File S3. (c) PC1 pattern was directly correlated with MEG power maps at 6 canonical frequency bands and intrinsic timescales. Consistent with the original findings, PC1 score was significantly correlated with hi-gamma power and intrinsic timescale (FDR-corrected). r_s denotes the Spearman's rank correlation coefficient.

bioRxiv preprint doi: https://doi.org/10.1101/2023.01.23.525101; this version posted January 23, 2023. The copyright holder for this preprint (which was not certified by peer review) is the author/funder, who has granted bioRxiv a license to display the preprint in perpetuity. It is made available under aCC-BY 4.0 International license.

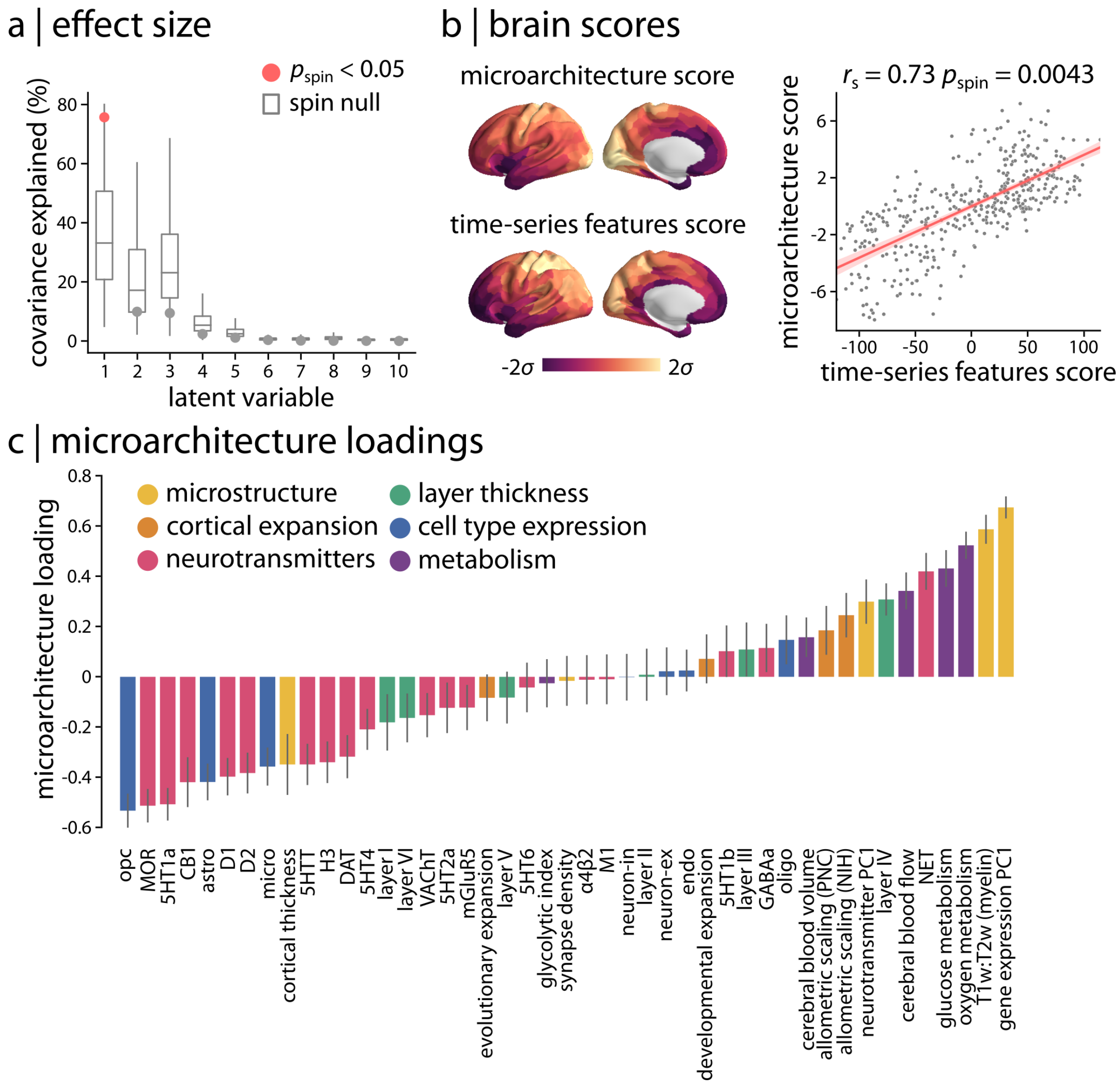

Figure S6. **Partial least squares (PLS) analysis for Schaefer-400** | PLS analysis was used to assess the multivariate relationship between micro-architectural and time-series features for the Schaefer-400 atlas. The results were consistent with the original findings for the Schaefer-100 atlas (Fig. 4). (a) PLS identified a single significant latent variable ($p_{\text{spin}} = 0.0083$, covariance explained $= 75.7\%$). (b) Spatial patterns of micro-architecture and time-series features scores are depicted for the first latent variable. The two brain score maps were significantly correlated, demonstrating similar patterns to the ones obtained for Schaefer-100 atlas (Fig. 4b). (c) Micro-architectural feature loadings were also consistent with the original findings (Fig. 4d). Full list of time-series feature loadings are included in the online Supplementary File S4. Consistent with the original analysis, the top loading features were mainly related to the linear correlation structure of the signal. r_s denotes the Spearman's rank correlation coefficient.

bioRxiv preprint doi: https://doi.org/10.1101/2023.01.23.525101; this version posted January 23, 2023. The copyright holder for this preprint (which was not certified by peer review) is the author/funder, who has granted bioRxiv a license to display the preprint in perpetuity. It is made available under aCC-BY 4.0 International license.

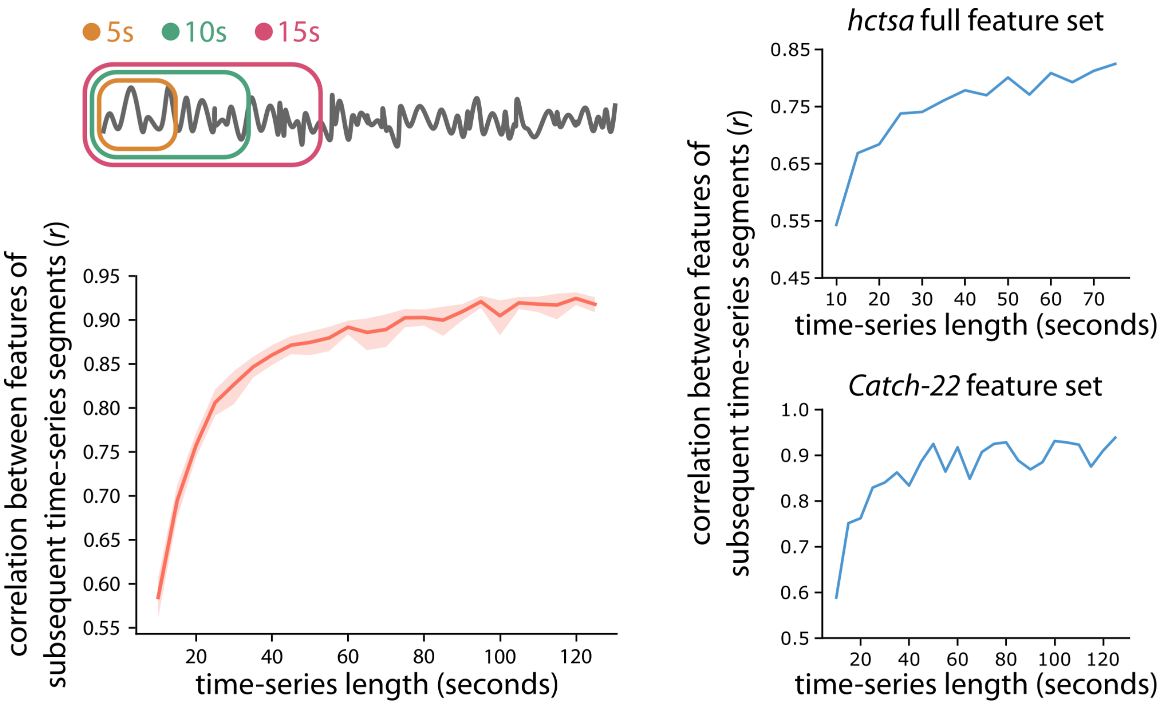

Figure S7. **Stability of time-series features |** To identify the time-series length required to robustly estimate the time-series features, we calculated a subset of `hctsa` features using the `catch-22` toolbox [115] on subsequent segments of time-series with varying length for each participant. We extracted time-series features from short segments of data ranging from 5 to 125 seconds in increments of 5 seconds. To identify the optimal time-series length required to estimate robust and stable features, we calculated the Pearson correlation coefficient r between features of two subsequent segments (e.g., features estimated from 10 and 5 seconds of data). The group-average correlation coefficient between the estimated features started to stabilize at time-series segments of around 30 seconds, consistent with previous reports [22] (left). To compare the stability analysis of `catch-22` features with full `hctsa` features, the correlation coefficients between subsequent segments of time-series are shown for a randomly selected participant (right).
